# RECRUITING FAMILY CAREGIVERS FOR INTERVENTIONS DESIGNED TO OPTIMIZE TRANSITIONS FROM HOSPITAL TO HOME

**DOI:** 10.1093/geroni/igae098.2112

**Published:** 2024-12-31

**Authors:** Allison Gustavson, Joan Griffin, Molly Horstman, Brystana Kaufman, Jay Mandrekar, Catherine Vanderboom, Cory Ingram, Diane Holland

**Affiliations:** Minneapolis Veterans Affairs Health Care System, Minneapolis, Minnesota, United States; Mayo Clinic, Rochester, Minnesota, United States; Baylor College of Medicine, Houston, Texas, United States; Duke University School of Medicine, Durham, North Carolina, United States; Mayo Clinic, Rochester, Minnesota, United States; Mayo Clinic, Rochester, Minnesota, United States; Mayo Clinic, Rochester, Minnesota, United States; Mayo Clinic, Rochester, Minnesota, United States

## Abstract

Recruiting family caregivers to participate in research, especially caregivers of people with severe or life limiting illnesses transitioning from hospital to home, is challenging. Traditional approaches for recruitment, including recruitment done on site at clinics or hospitals, became impracticable during the COVID-19 pandemic, leading to shifts toward virtual or remote recruitment and consent. In this presentation, Dr. Gustavson will compare the success of these two methodological approaches, in-person and remote recruitment and consent, used in the Technology-Enhanced Transitional Palliative Care (TPC) study. She found that the rate of recruitment and consent done in-person was 28% and the rate of remote recruitment and consent was 23%. Those recruited and consented remotely were more likely to be younger, white, have greater than high school education, and were children of or had another non-spousal relationship to the care recipient. She discusses the limitations and benefits of each recruitment modality, including the impact on representativeness of the study sample.

